# Unraveling the genetic links between obesity or insulin resistance and breast cancer through the impact of CD295 and ITLN1 SNPs with DNA damage in a case-controlled study with bioinformatics analysis

**DOI:** 10.3389/fmed.2025.1703759

**Published:** 2025-10-27

**Authors:** Nadia M. Hamdy, Yasser O. Mosaad, Reham Elshimy, Ahmad A. Hady, Queran Lin, Zayd Jastaniah, Razan Amjad, Huda Altoukhi, Hatim Almarzouki, Ahmed Abdelsamad, Hekmat M. El Magdoub, Doaa Fathi

**Affiliations:** ^1^Department of Biochemistry, Faculty of Pharmacy, Ain Shams University, Cairo, Egypt; ^2^Department of Pharmacy Practice and Clinical Pharmacy, Faculty of Pharmacy, Future University in Egypt (FUE), New Cairo, Egypt; ^3^Department of Clinical and Chemical Pathology, National Cancer Institute, Cairo University, Cairo, Egypt; ^4^Department of Clinical Oncology and Nuclear Medicine, Faculty of Medicine, Mansoura University, Mansoura, Egypt; ^5^Department of Primary Care and Public Health, Faculty of Medicine, WHO Collaborating Centre for Public Health Education and Training, School of Public Health, Imperial College London, London, United Kingdom; ^6^Department of Internal Medicine, Faculty of Medicine, King Abdulaziz University (KAU), Rabigh, Saudi Arabia; ^7^Radiotherapy Unit, Department of Radiology, King Abdulaziz University Hospital, Jeddah, Saudi Arabia; ^8^Department of Surgery II, University of Witten/Herdecke, Witten, Germany; ^9^Department of Oncological Surgery, Robotic SurgeryKnappschaft Vest-Hospital, Recklinghausen, Germany; ^10^Department of Biochemistry, Faculty of Pharmacy, Misr International University, Cairo, Egypt; ^11^Department of Biochemistry, Faculty of Pharmacy, Alexandria University, Alexandria, Egypt

**Keywords:** CD295, cytokines/inflammation, breast cancer (BC), polymorphism/mutations, ITLN1, obesity/insulin resistance (IR)/diabetes mellitus (DM), bioinformatics/*in silico*

## Abstract

**Background:**

Mutations in the cluster of differentiation (CD) 295 gene, which encodes a class I cytokine receptor, are associated with obesity and breast cancer (BC). Single-nucleotide polymorphisms (SNPs) in the adipocyte-inferred novel cytokine intelectin 1 (ITLN1) remain understudied in connection to CD295 polymorphisms and diabetes mellitus (DM) or a pre-diabetic state, as well as to DNA damage seen in BC.

**Aim:**

To explore whether CD295 (rs6700986) and ITLN1 (rs952804) SNPs impact BC with or without DM, insulin resistance (IR), or obesity. Effects of ITLN1 or CD295 polymorphism(s) on DNA damage in BC were also examined. All of these are to be confirmed by bioinformatics/*in silico* analysis.

**Subjects and methods:**

Blood samples from 170 women with BC (including 33 and 48 with DM and pre-diabetes, respectively) and from 108 age-matched women in the control group were collected. Plasma insulin, leptin, CD295, and ITLN1 levels were measured by ELISA. rs6700986 and rs952804 were analyzed by RT-PCR. DNA damage was assessed using the alkaline comet assay.

**Results:**

BC cases with clinical stage T II and positive LN, as well as tumor histologic grade III, presence of obesity, pre-diabetic events, DM, or IR, were associated with CD295 rs6700986 mutant homozygous (CC) and heterozygous (CT) genotypes and ITLN1 rs952804 mutant CT genotype (*p* ≤ 0.05). Tail DNA (%) and tail moment units were significantly associated with the CD295 rs6700986 CT and the ITLN1 rs952804 TT genotypes. The C allele (CT + CC vs. TT) and T allele (TT + CT vs. CC) for CD295 rs6700986 and ITLN1 rs952804, respectively, were associated with BC risk (*p* ≤ 0.05).

**Conclusion:**

CD295 (rs6700986) and ITLN1 (rs952804) SNPs should be considered as BC-associated susceptibility risk factors in obese, insulin resistance, or pre-diabetic individuals.

## Graphical abstract

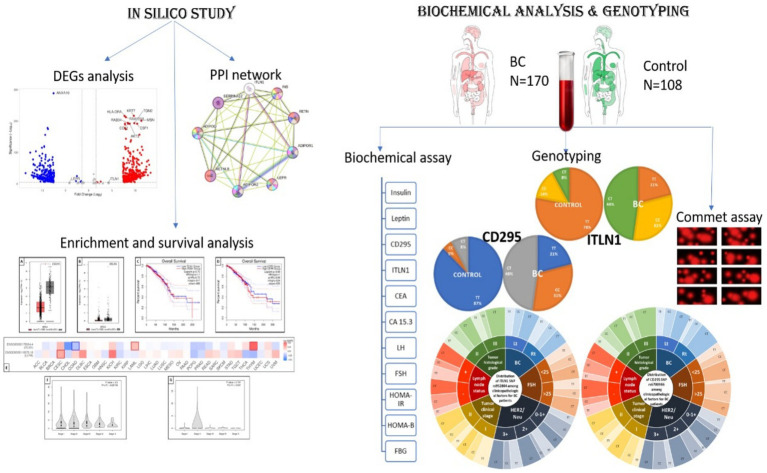

## 1 Introduction

Breast cancer (BC) is the most frequently diagnosed cancer among women worldwide (1) and in Egypt (2). Cancer cases, including BC, that have high levels of immune cells and/or elevated levels of pro-inflammatory cytokines are anticipated to have a poor prognosis (3–6) and if obese (7). Adipokines are proteins secreted by adipose tissue that are involved in a wide range of processes, including insulin sensitivity (8) and diseases (9–11). Meanwhile, adipocytokines, which are adipocyte-derived hormones, have also been implicated in the regulation of metabolism and insulin resistance (IR).

The protein encoded by the cluster of differentiation (CD) 295 gene belongs to a family of cytokine receptors. There are more than 50 cytokines in the adipokine family, including CD295, a class I cytokine receptor, which plays roles in malignancy in obese individuals (12). Long-chain CD295 receptor type b is responsible for most of the effects mediated by CD295 (13). CD295 is known to stimulate gene transcription by activating the cytosolic JAK–STAT proteins MAPK, PI3K, and AMPK (14). CD295 is also a receptor (R) for leptin (LEP) LEPR. According to the *in silico* database BioGRID^4.4^,^1^ it is involved in the cell surface receptor signaling pathway with transmembrane signaling receptor activity. CD295 is involved in the regulation of the superoxide metabolic process, angiogenesis, and the positive regulation of organ growth, as well as the positive regulation of mitochondrial membrane permeability, all of which may be implicated in cancer growth and progression^2^ from the Comparative Toxicogenomics Database (CTD).

The adipocyte-inferred cytokine intelectin 1 (ITLN1),^3^ according to the *in silico* database BioGRID^4.4^, it is a novel secretory and galactose-binding lectin that appears to have an anti-inflammatory role in both obesity and inflammation-related diseases, including BC (15). The ITLN1 gene product, omentin1, is mainly expressed in visceral adipose tissue and was shown to inhibit inflammatory responses and mitigate IR, as well as other obesity-related disorders (16) from CTD.^4^

An immediate relationship between obesity and IR and their related inflammatory states/oxidative stress to BC risk has been reported (17).

It is noteworthy to mention that hyperinsulinemia, in IR and obesity, triggers proliferative tissue anomalies due to the strong anabolic impact of insulin (INS), which stimulates DNA synthesis and cell proliferation (18). IR promotes elevated glucose with cytotoxic oxidative and pro-inflammatory milieu (45) that could be, definitely, associated with DNA strand break damage (19). DNA damage is a crucial hallmark in various cancer types (20).

Thus, the causes of CD295 and ITLN1 genes and their downstream protein products are numerous; therefore, if we detect genetic mutations/polymorphisms in these genes in BC cases with or without obesity, IR, or diabetes, this would direct the treatment plan for a better prognosis.

Single-nucleotide polymorphisms (SNPs), as one type of genetic variation, are related to various disease pathogenesis (21, 22). They are considered good prognostic indicators nowadays.

This study was conducted to explore whether SNPs in CD295 (ID rs6700986) and ITLN1 (rs952804) confer risk for metabolic derangements of IR and/or diabetes mellitus (DM) in female BC patients in Egypt. The first objective of this study was to explore how these SNPs affect BC in the presence or absence of diabetes, IR, or obesity. Whereas the second objective was to examine DNA damage in these patients using the comet test to detect DNA strand breaks in individual cells from mononuclear cells isolated from blood samples (23).

No study has examined the relationship between polymorphisms in these genes and the level of DNA damage in BC cases, so this will be the first clinical study to do this examination.

## 2 Subjects, materials, and methods

### 2.1 Sample size and study power

This study aims to investigate the role of some metabolic circulating genes/proteins in independent control and BC cases. Based on several previous studies testing CDs or protein receptors in BC or examining SNPs in BC where their expression was normally distributed with a standard deviation of more than 2.5 and a large effect size of more than (1.0). Additionally, the calculation was confirmed based on the odds ratio (OR) from independent samples in a genetic mutation study using Fisher’s exact test. The *α*-error level was fixed at 0.05, the power was set at 80%, and the case–control ratio was set at 1.

If the true difference in the BC group and control group means is 2.25, we will need to study 25 experimental subjects and 25 control subjects to reject the null hypothesis that the population means of the experimental and control groups are equal with probability (power) 0.8. The Type I error probability associated with this test of the null hypothesis is 0.05. The sample size will be 100 cases; this number will be increased by 15% for expected losses.

The sample size calculation was done using PS: Power and Sample Size Calculations software, version 3.0.11 for MS Windows (William D. Dupont and Walton D., Vanderbilt University, Nashville, Tennessee, United States).

### 2.2 Subjects

The study protocol was approved by the ethical committees of the Faculty of Pharmacy, Ain Shams University (code number 30/7/16-F-EBD-01-03), and the National Cancer Institute (NCI) (code number IRB00004025) at Cairo University, Cairo, Egypt. The study was carried out in accordance with the regulations and recommendations of the Declaration of Helsinki. Written informed consent (IC) was obtained from all study subjects.

#### 2.2.1 Inclusion criteria

Females diagnosed with invasive ductal carcinoma (IDC) for the first time at age 43 or older were included. A total of 170 postmenopausal female patients aged 43–75 were selected, and the median age was 53 years.

#### 2.2.2 Exclusion criteria

Female BC patients having incomplete data or histopathologic diagnosis, as well as patients with metastasis at the time of initial diagnosis, were excluded. Other exclusion criteria included a previous or current history of acute or chronic viral hepatitis, malignant disease, acute infections, pituitary, adrenal, thyroid, or pancreatic disease, or evidence of any other endocrine disorders or prolonged use of corticosteroids or sex hormones.

#### 2.2.3 Control group

A group of 108 age-matched healthy female controls (median age 53 years) with normal liver enzyme levels and no clinical or laboratory evidence of BC was recruited during routine wellness examinations.

#### 2.2.4 Participant data

The demographic characteristics of the study subjects are summarized in Table 1.

**Table 1:** Anthropometric parameters; obesity and diabetes factors, and BC clinical factors in BC patients compared to the control group.

Groups	Control	BC patients	
*n*	108	170	*P*-value
Parameter			
Age (years)	53 (43–66)	53 (43–75)	NS
INS (μIU/mL)	8 (4.5–19)	10.6 (3.8–17.3)	**0.007**
FBG (mg/dL)	100 (66–144)	120 (85–224)	NS
HOMA-IR index	2.2 (0.81–4.9)	3.4 (0.9–6.6)	**0.02**
HOMA-B	0.8 (0.31–6)	0.68 (0.13–1.94)	**0.03**
Obese (−/+)	40/68	38/132	NS
IR (−/+)	50/58	12/158	NS
Diabetes (−/pre/+)	90/16/2	88/48/33	NS
Leptin (ng/mL)	7 (2–11)	15 (8–50)	**<0.001**
LepR (ng/mL)	22 (11–27)	20 (13–27)	**0.001**
ITLN1 (ng/mL)	99 (66–177)	40 (30–66)	**<0.001**
Ejection fraction %		64 (54–80)	
CEA (ng/mL)		4 (1–10)	
CA15.3 (U/mL)		20 (1–126)	
Ki-67		70 (15–80)	
LH (IU/L)^a^		9.9 ± 0.12	
FSH (mIU/L)^a^		24.5 ± 1.85	
Estradiol (pg/mL)^a^		27.1 ± 6.9	
Tumor clinical stage (T) (I/II) (n)		69/101	
Lymph node status (N) (−/+) (n)		66/104	
Metastasis (M) (−/+) (n)		170/0	
Tumor histological grades (II/III) (n)		141/29	
BIRAD (III/IV) (n)		42/128	

Statistical analyses were conducted using the SPSS statistical package for Windows Version 22.0 (SPSS Software, Chicago, IL, United States). Data are presented as median (range). Statistical analysis was performed using the Mann–Whitney U test. ^a^Data are presented as mean ± S.D. Statistical analysis was performed using a chi-square test. FBG, fasting blood glucose; INS, insulin; HOMA-IR, homeostasis model assessment-insulin resistance; CEA, carcinoembryonic antigen; CA 15.3, cancer antigen 15.3; LH, luteinizing hormone; FSH, follicle-stimulating hormone; NS, non-significant; n, number. All bold numbers indicate significant *p*-value.

Clinical data were obtained from medical records and original pathology reports and were compiled in an Excel database (Microsoft Corporation, Redmond, WA, United States). The following parameters were assessed: patient age, tumor size (defined as sonographic diameter (mm) on diagnosis), initial tumor stage and nodal status according to tumor-metastasis-node (TMN) classification, histologic subtype, estrogen receptor (ER) status, progesterone receptor (PR) status, human epidermal receptor 2 (HER2) status (a score of 0 to +1 was regarded as HER2 negative and a score of +2 is positive), grading and proliferation status as assessed by Ki-67 staining, and chemotherapy regimen (doxorubicin/trastuzumab/lapatinib). The BI-RADS (Breast Imaging-Reporting and Data System) risk assessment and quality assurance tool, developed by the American College of Radiology, is defined for all patients as ranging from 1 (nothing) to 4 (malignancy).

All histopathological parameters included were derived from the original pathology reports. Scoring was performed by a specialized pathologist at the Pathology Department, NCI, Cairo University, in accordance with standardized protocols.

### 2.3 Study design

This is a descriptive, observational, and case-controlled study.

### 2.4 Blood sample collection and preparation

A 7 mL blood sample was collected from each patient, after an 8-h fast, into two K_3_EDTA Vacutainer tubes at the end of the clinical examination interview. One tube was used to prepare plasma from 3 mL of blood by centrifuging at 4,000 rpm for 15 min. Plasma samples were aliquoted and stored at −80°C until biochemical assessment (ELISA). One part, 3 mL of the sample, was used for DNA extraction from the blood, which was carried out using the QIAamp DNA Mini Kit protocol (QIAGEN, Inc., Thermo Fisher Scientific, Santa Clarita, 3,380 Central Expy, Santa Clara, CA95051, United States). The extracted DNA was stored at −80 C until it was used for SNP assays. The remaining sample was used for the alkaline comet assays.

#### 2.4.1 Biochemical analysis

MyBioSource human ELISA was used to determine plasma (fasting) INS (catalog #MBS024180), leptin (catalog #MBS2020720), CD295 (catalog #MBS355370), and ITLN1 (catalog # MBS763109) levels as well as carcinoembryonic antigen (CEA) (catalog #MBS2020244), cancer antigen 15.3 (CA15.3) (catalog #MBS161810), luteinizing hormone (LH) (catalog #MBS047228), and follicle stimulating hormone (FSH) (catalog #MBS705942) according to the manufacturers’ instructions run in duplicates.

Homeostasis model assessment (HOMA), a measure of IR, was calculated as [(Fasting blood glucose (FBG) × fasting INS)/22.5] (24). FBG was recorded from patients’ sheets. This value is regarded as a simple, inexpensive, and reliable surrogate measure of IR, whereas the HOMA of *β*-cell function (HOMA-B) index, computed as the product of 20 and basal INS levels divided by the value of basal glucose concentrations minus 3.5, is accepted as a good measure of β-cell function (25).

#### 2.4.2 Genetic analysis

Genotyping was performed using Assays-by-Design supplied by Applied Biosystems International (ABI) (Applied Biosystems, 850 Lincoln Centre Dr., Foster City, CA 94404, United States). Reactions were performed on a 7500 ABI Sequence Detection System.

##### 2.4.2.1 SNPs choice

SNPs^5^ were chosen based on a minor allele frequency that exceeded 5%.

The CD295 rs6700986 polymorphism is a T/C (FWD) single-nucleotide variation on human chromosome 1:180,452,403, p31.3, Ancestral Allele: C. GAGAATCACTTGAAACCTAGGAGGC[T/C]AAGGTTGTGGTGAGCCGAGATCATG, Gene: ACBD6 (GeneView), Functional Consequence: intron variant, Global MAF: T = 0.4603/2305.

The ITLN1 rs952804 polymorphism is a C/T (FWD) single-nucleotide variation on human chromosome 1:160,874,955, q23.3, Ancestral Allele: C. CCACCTGCAGCTTTAGAATTGGGTT[C/T]ATCTGTCTTCTCTATCACTTCTTTA, Global MAF: T = 0.4335/2171.

##### 2.4.2.2 Genotyping and single-nucleotide polymorphism

SNP genotyping was performed using the TaqMan^®^ method for allele-specific detection (ABI), which involves real-time polymerase chain reaction (PCR) amplification with fluorescence detection (26). The experiments were performed using an ABI Prism^®^ Sequence Detection System (ABI) and TaqMan^®^ Universal PCR Master Mix (ABI) together with a 20 ng genomic DNA template (10 ng/μL). Each 25 μL reaction volume contained 100 ng genomic DNA, 0.2 mM dNTPs, 20 mM Tris–HCl (pH 8.8), 10 mM KCl, 10 mM (NH_4_)_2_SO_4_, 2 mM MgSO_4_, 0.1% Triton X-100, and 1-unit Taq polymerase (New England BioLabs (NEB), 240 County Rd., Ipswich, Massachusetts 01938, United States). There was 0% missing data, no duplicate concordance, and no samples were excluded.

PCR amplification conditions are as follows: an initial denaturation step at 94°C for 5 min, followed by 34 cycles of 30 s at 94 C, 45 s at 60 C, and 45 s at 72 C, and a final elongation step at 72°C for 10 min. After PCR amplification, we performed an endpoint plate read using an ABI StepOne Plus Real-Time PCR System from Fischer Scientific (Thermo Fischer Scientific, United States).

#### 2.4.3 Comet assay

Alkaline comet slide assays were performed according to a standard protocol (27). Comet assay kit (Trevigen, 8,405 Helgerman Court, Gaithersburg, 20,877 Maryland, United States).

To the coated slide, not more than 10 μL of cell suspension in phosphate-buffered saline (PBS) containing about 10^4^ cells was mixed with 70 μL of 0.6% low-melting-point agarose (LMPA by Sigma CAS No: 39346–81-1). Placed the coverslip and put the slide on a slide tray (by ABDOS Life Sciences), resting on ice packs until the agarose layer hardened (~5–10 min). Gently coverslipped, slid off, and added a third agarose layer (80 μL LMPA) to the slide. Replaced the coverslip and returned the slide to the tray until the agarose layer hardened (5–10 min). The slides were placed in an electrophoresis chamber containing 0.3 M NaOH, 1 mM Na2EDTA buffer, devoid of SDS (pH 13) for 40 min. The electrophoresis conditions were 2 V/cm for 2 min. and 100 mA. Staining with ethidium bromide 20 μg/mL at 4°C. The observation was made with the samples still humid, and the DNA fragment migration patterns of 100 cells for each dose level were evaluated with a fluorescence microscope [with excitation filter 420–490 nm (issue 510 nm)]. The comet’s tail lengths were measured from the middle of the nucleus to the end of the tail with a 40x increase for the count, and to measure the size of the comet. For visualization of DNA damage, observations are made of ethidium bromide-stained DNA using a 40x objective on a fluorescent microscope. All slides were duplicated.

DNA migration in peripheral blood leukocytes was measured using a computer-based image analysis system (COMET IV software, Life Science Software, Instem, West Conshohocken, 161 Washington St, Conshohocken, Pennsylvania 19,428, United States)^6^ connected to a camera (Leica DFC340 FX camera by Leica Microsystems, Ernst-Leitz-Straße 17–37, 35578, Wetzlar, Germany). The supplied software computed all major measurement parameters, including tail length, which describes the distance from the center of the nucleoid mass to the distal tail end, tail intensity (TI): the relative fluorescence intensity of the comet tail that is a measure of the percentage of DNA in the tail, and tail moment unit (TMU): essentially the product of tail length and TI.

### 2.5 Bioinformatics/*in silico* analysis (accessed May 2024 and rechecked May 2025)

#### 2.5.1 Identification of differentially expressed genes in breast cancer

To investigate molecular alterations in BC cells relative to normal cells, differentially expressed genes (DEGs) were identified at the transcriptomic level. Gene expression datasets were obtained from the EMBL-EBI Expression Atlas^7^ and subsequently analyzed using the VolcaNoseR web application,^8^ which enables interactive visualization and exploration of volcano plots to assess both the magnitude and statistical significance of gene expression changes. Moreover, the Xena Functional Genomics Explorer for Differential Gene Expression Analysis was used^9^ (28) by the University of California, Santa Cruz (UCSC). Visualization and quality control are also performed using Principal Component Analysis (PCA) (29) to identify global patterns in high-dimensional datasets. Gene expression values were transformed into principal components (PCs), a set of linearly uncorrelated features that represent the most relevant sources of variance in the data, and subsequently visualized using a scatter plot.

#### 2.5.2 Protein–protein interaction network

To investigate the interactions between studied proteins, we constructed a protein–protein interaction (PPI) network using the STRING database^10^ to study their interactions with other important proteins and pathways.

#### 2.5.3 Enrichment analysis and gene-disease association

To identify the cellular pathways associated with our target genes and gene-disease association, the Enrichr-KG database was accessed at https://maayanlab.cloud/enrichr-kg.

#### 2.5.4 Genotype-tissue expression and genotype survival analysis

Genotype-tissue expression and genotype survival analysis were conducted using the Gene Expression Profiling Interactive Analysis (GEPIA) tool.^11^

### 2.6 Statistical analysis

All statistical analyses were conducted using the Statistical Package for the Social Sciences (SPSS) for Windows Version 22.0 (SPSS Software, Chicago, IL, United States) and the R language (30). First, we tested the normal distribution of the data using the Shapiro–Wilk Test Calculator.^12^ The results for continuous variables were expressed as mean ± standard deviation (SD), except for the median (range) for nonparametric data. To study the association between variables, an OR was calculated. For categorical variables, a chi-square test was used to compare differences between different groups. The best cutoff values for the investigated parameter(s) (FSH) were calculated from receiver operating characteristic (ROC) curves. Genetic analyses were also conducted using unconditional logistic regression. Initially, we used univariate analyses to examine possible associations between each polymorphism and cancer grades (II and III) or T (I and II) or N (−/+), or DNA damage and associated metabolic factors (e.g., INS, HOMA-IR, and IR). We additionally conducted subgroup analyses to examine the potential interactions between the levels of the pre-specified factors and the studied SNPs. Based on the findings from our population, we computed the frequencies of each allele using the Hardy–Weinberg equilibrium (HWE) online software.^13^ A likelihood ratio test was performed to test the significance. Correlations were assessed using the Spearman correlation coefficient (r).

Moreover, a PERMANOVA (Permutational Multivariate Analysis of Variance) non-parametric statistical test for applied multivariate group statistics comparisons, in R, without assumptions of normality, compares groups based on a chosen dissimilarity measure, rather than means. If a significant separation (*p* < 0.05) between BC cases and controls exists in multivariate space, this would demonstrate that the combined metabolic and genetic profile creates distinct patient phenotypes. The pseudo-F statistic was used to measure the ratio of between-group to within-group variation in multivariate space. A significant result indicates that BC patients occupy a different region of the metabolic-genetic landscape compared to healthy controls. This finding supports the concept that BC involves systemic metabolic alterations rather than isolated changes in biomarkers. A Sankey diagram was used to show the output of a PERMANOVA analysis.

Two-tailed analyses were performed, and *p*-values ≤ 0.05 were considered statistically significant.

## 3 Results

A total of 278 individuals were recruited for this study, including 170 females with BC, of whom 158 were insulin resistant (Table 1). Among the BC patients, 132 were obese. Of the obese BC patients, 88 were normoglycemic, 48 were pre-diabetic, and 33 had DM. The healthy control group had 108 individuals, of whom 40 and 68 were lean and obese, respectively (Table 1).

### 3.1 Clinical and biological analysis of the study population

Various anthropometric parameters, obesity and diabetes factors, and BC clinical measures, as well as plasma levels of leptin, CD295, and ITLN1, were collected (Table 1), as was information concerning the SNPs under study and DNA damage parameters for both the BC patients and the healthy controls (Table 2).

**Table 2:** Studied SNPs and DNA damage parameters of BC patients compared to the control group.

Groups		Control	BC	
n		108	170	*P*-value
Parameters				
CD295 rs6700986 ^a^	Genotype			
	Homozygous wild-type TT	94	36	**0.04**
	Homozygous mutant-type CC	5	53	**<0.001**
	Heterozygous mutant-type CT	9	81	**0.007**
	CC + CT	14	134	**<0.001**
ITLN1 rs952804^a^	Genotype			
	Homozygous wild-type CC	84	46	**0.037**
	Homozygous mutant-type TT	15	48	**0.001**
	Heterozygous mutant-type CT	9	76	**<0.001**
	TT + CT	24	124	**<0.001**
DNA damage	Tail intensity (%)	2 (1–4)	13 (8–19)	**0.001**
	Tail length (μm)	0.7 (0.53–0.86)	3.5 (2.7–4.34)	**0.001**
	Tail DNA (%)	1.3 (0.9–1.84)	3.95 (2.45–4.97)	**0.02**
	Tail moment unit	3 (2.4–5.4)	12 (9.27–17)	**0.001**

Statistical analyses were conducted using the SPSS statistical package for Windows Version 22.0 (SPSS Software, Chicago, IL, United States). Data are presented as median (range). Statistical analysis was performed using the Mann–Whitney U test. ^a^Discrete data are given as numbers, and statistical analysis was performed using a chi-square test. ITLN1, intelectin-1; CD295, cluster of differentiation 295; n, number. All bold numbers indicate significant *p*-value.

### 3.2 Distribution of CD295 and ITLN1 genotypes

There were significant differences among the measured values between the control and BC group in terms of polymorphism frequency and DNA damage (see below; *p* ≤ 0.05) (Table 2). The CD295 wild homozygous genotype (TT) was detected in almost all controls (94/108; 87%), but only 36 (21%) BC patients carried this genotype. Meanwhile, mutant homozygous (CC) and heterozygous (CT) CD295 genotypes were observed in 53 (31.1%) and 81 (47.6%) BC cases, respectively. For ITLN1, the wild homozygous genotype (CC) was detected in the majority of the control subjects (77.7%), whereas 46 (43%) of BC patients carried this genotype (Table 2). Mutant homozygous (TT) and CT genotypes were reported in 48 (28.2%) and 76 (44.7%) BC cases, respectively.

**Figure 1:**
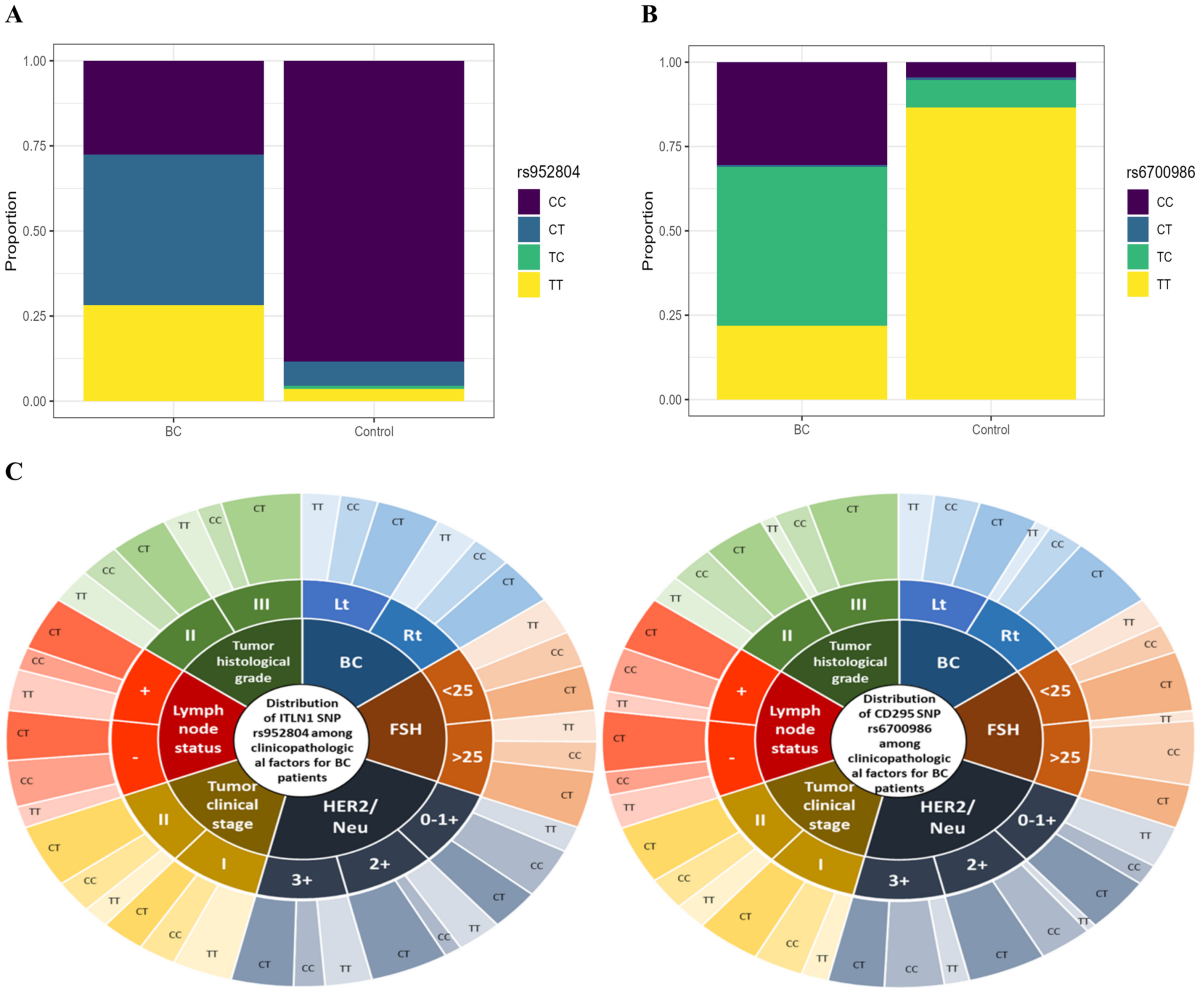
Distribution of ITLN1 SNP rs952804 and CD295 SNP rs6700986; ITLN1 SNP rs952804 **(A)** and CD295 SNP rs6700986 **(B)** stacked bar charts, respectively, among BC patients and control group (The Wilcoxon rank-sum tests provide non-parametric comparisons of biomarker distributions between BC cases and controls), and **(C)** ITLN1 SNP rs952804 and CD295 SNP rs6700986 genotypes donut figure among clinicopathological factors for BC patients (*n* = 170); the donut figure is created in excel.

SNPs data presented in Table 2 are visualized in Figures 1A,B as well. The chi-squared tests revealed highly significant associations between both genetic variants and BC risk. For rs6700986 (LEPR gene) and rs952804 (ITLN1 gene), both showed *p* < 2e-16, indicating extremely strong evidence against the null hypothesis of no association. The genotype frequency patterns are particularly striking: rs6700986 shows the protective TT genotype enriched in controls (~90% vs. ~25% in cases), while risk-associated C-containing genotypes (TC, CT, CC) predominate in BC patients. Similarly, rs952804 demonstrates CC genotypes as protective in controls (~80% vs. ~20% in cases), with T-containing genotypes elevated in BC cases.

### 3.3 Distribution of the studied SNPs genotypes among the BC patient clinicopathological factors

Stratification of the BC group according to FSH (mIU/L) values revealed that the CD295 rs6700986 CC and CT genotypes were significantly associated (*p* ≤ 0.05) with BC cases that had FSH values > 25 mIU/L. A similar association was seen for BC patients who were CT for the ITLN1 rs952804 mutation (Table 3). HER2 cancer cases with a + 3 score had a significantly higher frequency of CC and CT CD295 rs6700986 genotypes compared to those who had the wild homozygous (TT) genotype (p ≤ 0.05). Similar results were also seen for ITLN1 rs952804 mutant CT cases.

**Table 3:** Distribution of CD295 SNP rs6700986 and ITLN1 SNP rs952804 genotypes among clinicopathological factors for BC patients (*n* = 170).

SNP		CD295 rs6700986				ITLN1 rs952804			
		TT	CC	CT	*P*-value*,**	CC	CT	TT	*P*-value***
Clinicopathological factors									
Breast cancer	Lt	31 (25.2)	40 (32.5)	52 (42.3)	**0.035**	33 (26.8)	56 (45.5)	34 (27.6)	NS
	Rt	5 (10.9)	12 (26.1)	29 (63)	NS	13 (28.3)	19 (41.3)	14 (30.4)	NS
FSH (mIU/L)	< 25	33 (49.3)	33 (49)	68 (51)	NS	18 (30)	23 (38.3)	19 (31.6)	NS
	> 25	3 (8.6)	19 (54.3)	13 (37)	**0.014**	28 (25.7)	52 (47.7)	29 (26.6)	**0.016**
HER2	0–1+	24 (35.3)	12 (17.6)	32 (47.1)	NS	29 (42.7)	24 (35.3)	15 (22.1)	NS
	2+	4 (7.1)	21 (37.5)	31 (55.4)	NS	7 (12.5)	31 (55.4)	18 (32.1)	NS
	3+	8 (17.8)	19 (42.2)	18 (40)	**0.001**	10 (22.2)	20 (44.4)	15 (33.3)	**0.004**
Tumor clinical stage (T)	I	13 (19.1)	24 (35.3)	31 (45.6)	NS	18 (26.5)	21 (30.9)	29 (42.6)	NS
	II	23 (22.8)	28 (27.7)	50 (49.5)	NS	28 (27.7)	54 (53.5)	19 (18.8)	**0.002**
Lymph node status (N)	−	19 (28.8)	13 (19.7)	34 (51.5)	NS	26 (39.4)	28 (42.4)	12 (18.2)	NS
	+	17 (16.5)	39 (37.9)	47 (45.6)	**0.024**	20 (19.4)	47 (45.6)	36 (35)	0.007*
Tumor histological grade	II	33 (23.4)	45 (31.9)	63 (44.7)	NS	41 (29.1)	59 (41.8)	41 (29.1)	NS
	III	3 (10.7)	7 (25)	18 (64.3)	0.032**	5 (17.9)	16 (57.1)	7 (25)	**0.027**

Statistical analyses were conducted using the SPSS statistical package for Windows Version 22.0 (SPSS Software, Chicago, IL, United States). Data are numbers (%), significant at (*P* ≤ 0.05) using a chi-square test. For FSH, the best cutoff value (=25) was calculated by the ROC curve. *CC different from TT, **CT different from TT, ***CT different from CC. ITLN1, intelectin-1; FSH, follicle-stimulating hormone; CD295, cluster of differentiation 295, NS, non-significant. All bold numbers indicate significant *p*-value.

Tumor clinical stage T II and positive LN involvement, as well as tumor histologic grade III, were associated with CD295 rs6700986 CC and CT genotypes, as well as ITLN1 rs952804 mutant CT cases (*p* ≤ 0.05) (Table 3). All the data presented in Table 3 are also visualized in Figure 1C.

### 3.4 Distribution of CD295 rs6700986 and ITLN1 rs95280 genotype variants among BC patient obesity/IR factors

The presence of obesity, IR, or DM, as well as having experienced a pre-diabetic event, was associated with CC (for obesity and IR only) and CT CD295 rs6700986 genotypes (*p* ≤ 0.05) (Table 4). Furthermore, obesity, pre-diabetic event, and being diabetic, along with IR, were associated with BC cases that were ITLN1 rs952804 mutant CT (*p* ≤ 0.05) (Table 4).

**Table 4:** Distribution of genotype variants of CD295 rs6700986 and ITLN1 rs952804 SNPs among obesity/IR factors in BC patients (*n* = 170).

SNP		CD295 rs6700986				ITLN1 rs952804			
		TT	CC	CT	*P*-value*	CC	CT	TT	*P*-value***
Parameters	Event								
Obesity	(−)	8 (21.1)	6 (15.8)	24 (63.2)	NS	11 (28.9)	18 (47.4)	9 (23.7)	NS
	(+)	28 (21.4)	46 (35.1)	58 (43.5)	0.05**	35 (26.7)	57 (43.5)	40 (29.8)	**0.05**
Diabetes	(−)	20 (22.7)	27 (30.7)	41 (46.6)	NS	27 (30.7)	32 (36.4)	29 (33)	NS
	(Pre)	9 (18.8)	14 (29.2)	25 (52.1)	**0.05**	11 (22.9)	23 (47.9)	14 (29.2)	**0.03**
	(+)	7 (21.2)	11 (33.3)	15 (45.5)	**0.04**	8 (24.2)	20 (60.6)	5 (15.2)	**0.045**
IR	(−)	10 (90.9)	1 (9.1)	0 (0)	NS	11 (100)	0 (0)	0 (0)	NS
	(+)	26 (16.5)	51 (32.3)	81 (51.3)	0.01**	35 (22.2)	75 (47.5)	48 (30.4)	**0.01**

Statistical analyses were conducted using the SPSS statistical package for Windows Version 22.0 (SPSS Software, Chicago, IL, United States). Data are numbers (%), significant at (*P* ≤ 0.05) using a chi-square test. * CT different from TT, ** CC different from TT, *** CT different from CC. IR, insulin resistance; NS, non-significant. All bold numbers indicate significant *p*-value.

### 3.5 Association between CD295 rs6700986 and ITLN1 rs952804 polymorphism and anthropometric parameters of BC patients

Mutant homozygous (CC) and CT genotypes for CD295 rs6700986 and ITLN1 rs952804 mutant homozygous (TT) and CT cases were significantly (*p* ≤ 0.05) associated with higher age (median 54 and 53, respectively) for both SNPs tested (Table 5). This association also occurred for BC patients who had decreased plasma CEA (ng/mL) levels, increased insulin (μIU/mL) levels, IR, or increased plasma leptin (ng/mL) levels (Table 5).

**Table 5:** Association between CD295 rs6700986 and ITLN1rs952804 polymorphisms and anthropometric parameters in BC patients (*n* = 170).

SNP	CD295 rs6700986				ITLN1 rs952804			
	TT	CC	CT	*P*-value*,**	CC	CT	TT	*P*-value**,***
Parameters								
Age (years)	50 (47–64)	54 (43–75)	53 (46–75)	**0.05**	50 (47–75)	53 (45–75)	54 (43–75)	0.001***
CEA (ng/mL)	4.5 (1–10)	3.5 (1–10)	4 (1–10)	**0.029**	9 (1–10)	4 (1–10)	3.8 (1–10)	**0.003**
Insulin (μIU/mL)	7.2 (3.8–16.1)	13.7 (4.9–17.3)	10.4 (5.1–15.8)	**0.001**	8.1 (3.8–13.1)	10.4 (6–15.8)	14.4 (9.5–17.3)	**0.001**
HOMA-IR	2.4 (0.9–4.5)	3.8 (1.2–6.6)	3.3 (2.5–4.3)	**0.001**	2.5 (0.9–3.2)	3.4 (2.7–4.1)	4 (3.4–6.6)	**0.0001**
Leptin (ng/mL)	15 (8–19)	16 (11–50)	15 (8–20)	0.003**	15 (8–20)	15 (8–50)	16 (11–25)	0.015**

Statistical analyses were conducted using the SPSS statistical package for Windows Version 22.0 (SPSS Software, Chicago, IL, United States). Data are presented as median (range), with significance at *P* ≤ 0.05 using a chi-square test. *CT different from TT, **CC different from TT, ***CT different from CC. HOMA-IR; homeostasis model assessment-insulin resistance, ITLN1, intelectin-1; CEA, carcinoembryonic antigen; CD295, cluster of differentiation 295. All bold numbers indicate significant *p*-value.

**Figure 2:**
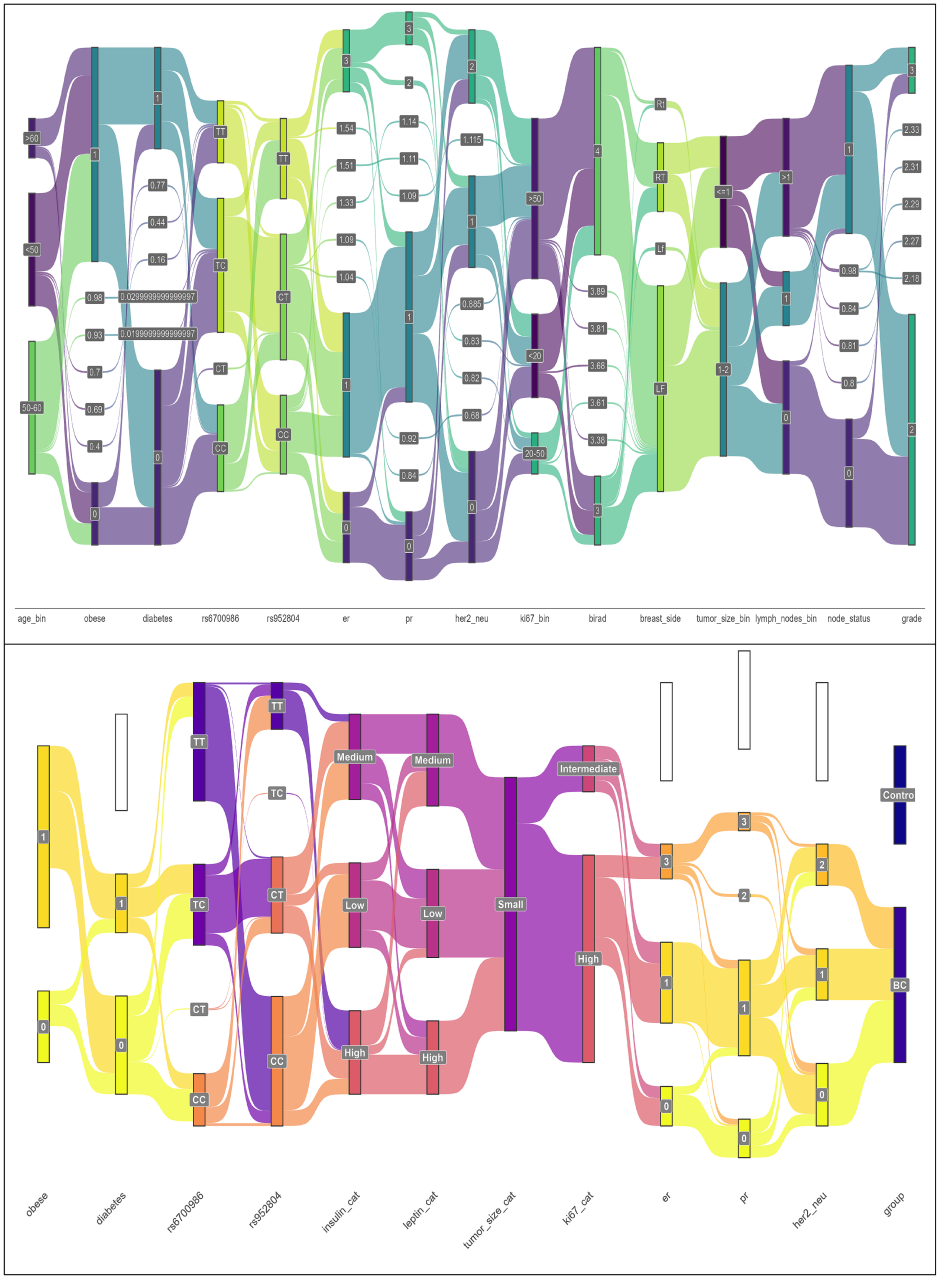
Sankey plots for the association of CD295 and ITLN1 SNPs and progression markers in BC patients’ group (*n* = 170) (Sankey plots showed the output of a PERMANOVA analysis).

Collectively, a Sankey diagram is developed to illustrate the association of CD295 and ITLN1 SNPs and progression markers in BC, as presented in Figure 2. The Sankey diagram illustrates the proportional patient flows through risk factor combinations, revealing dominant pathways from genetic predisposition to metabolic dysfunction and ultimately to cancer development.

**Figure 3:**
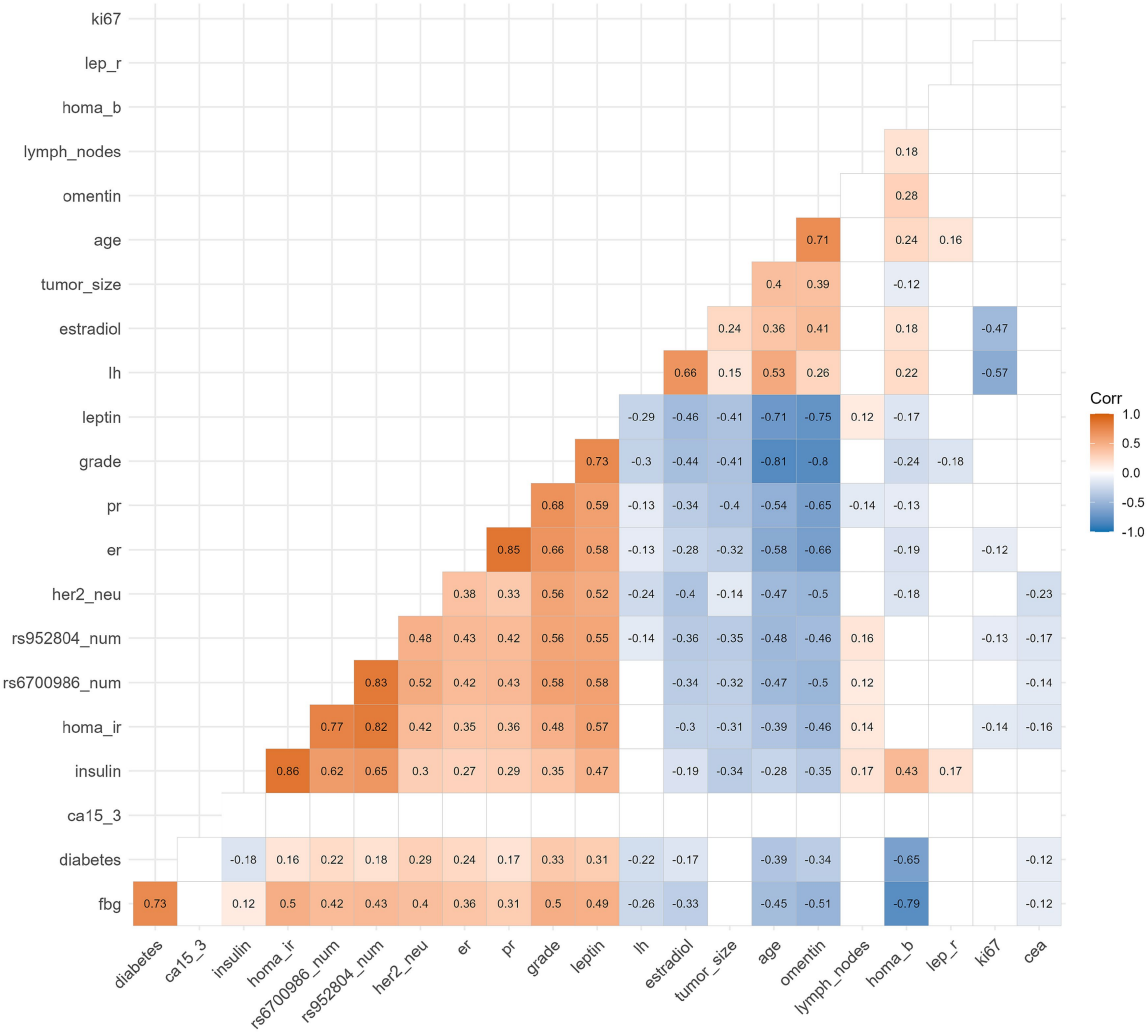
Correlation heatmap of clinical, biological, and genetic features (SNPs) in the BC patients. Following established correlation interpretation criteria, correlations above 0.4 (light color) indicate moderate associations, while those above 0.6 (dark color) represent strong relationships.

In Figure 3, the heatmap for Spearman correlations reveals interconnected metabolic networks with several statistically significant relationships. A negative correlation was observed between ITLN1 and tumor grade and a positive correlation with age. Positive correlations were observed between the HOMA-IR and ITLN1 and CD295 SNPs, as well as a positive correlation between LEP and tumor grade, establishing connections between genetic variants and metabolic phenotypes.

### 3.6 Relationship between studied SNPs CD295 rs6700986 and ITLN1 rs952804 genotypes and DNA damage parameters

Among DNA damage parameters, tail DNA (%) and tail moment unit were significantly (*p* ≤ 0.05) associated with CD295 rs6700986 mutant CT genotype and ITLN1 rs952804 mutant homozygous (TT) genotype (Table 6).

**Table 6:** Genotype distribution of CD295 rs6700986 and ITLN1 rs952804 SNPs relative to DNA damage parameters in BC patients (*n* = 170).

SNP	CD295 rs6700986				ITLN1 rs952804			
	TT	CC	CT	*P*-value*	CC	CT	TT	*P*-value**
DNA damage								
Tailed (%)	13 (8–19)	13 (8–17)	13 (8–19)	NS	13 (8–19)	13 (8–19)	13 (8–17)	NS
Tail length (μm)	3.8 (2.7–4.3)	3.4 (3–4.3)	3.5 (2.7–4.3)	NS	3.6 (2.7–4.3)	3.6 (2.7–4.3)	3.5 (3–4.3)	NS
Tail DNA (%)	3.7 (2.5–5)	4 (2.5–5)	3.5 (2.5–5)	**0.043**	4 (2.5–5)	4 (2.5–5)	3.4 (2.5–5)	**0.029**
Tail moment unit	12.2 (9.3–17)	12.2 (10–17.1)	11.6 (9.3–17)	**0.031**	12.2 (10–17.1)	12.2 (10–17.1)	11 (9.3–16.1)	**0.016**

Statistical analyses were conducted using the SPSS statistical package for Windows Version 22.0 (SPSS Software, Chicago, IL, United States). Data are presented as median (range), with significance at *P* ≤ 0.05 using a chi-square test. *CT different from TT, **TT different from CC. NS, non-significant. All bold numbers indicate significant *p*-value.

### 3.7 Bioinformatics analysis results

#### 3.7.1 Identification of CD295 and ITLN1 in breast cancer DEGs

**Figure 4:**
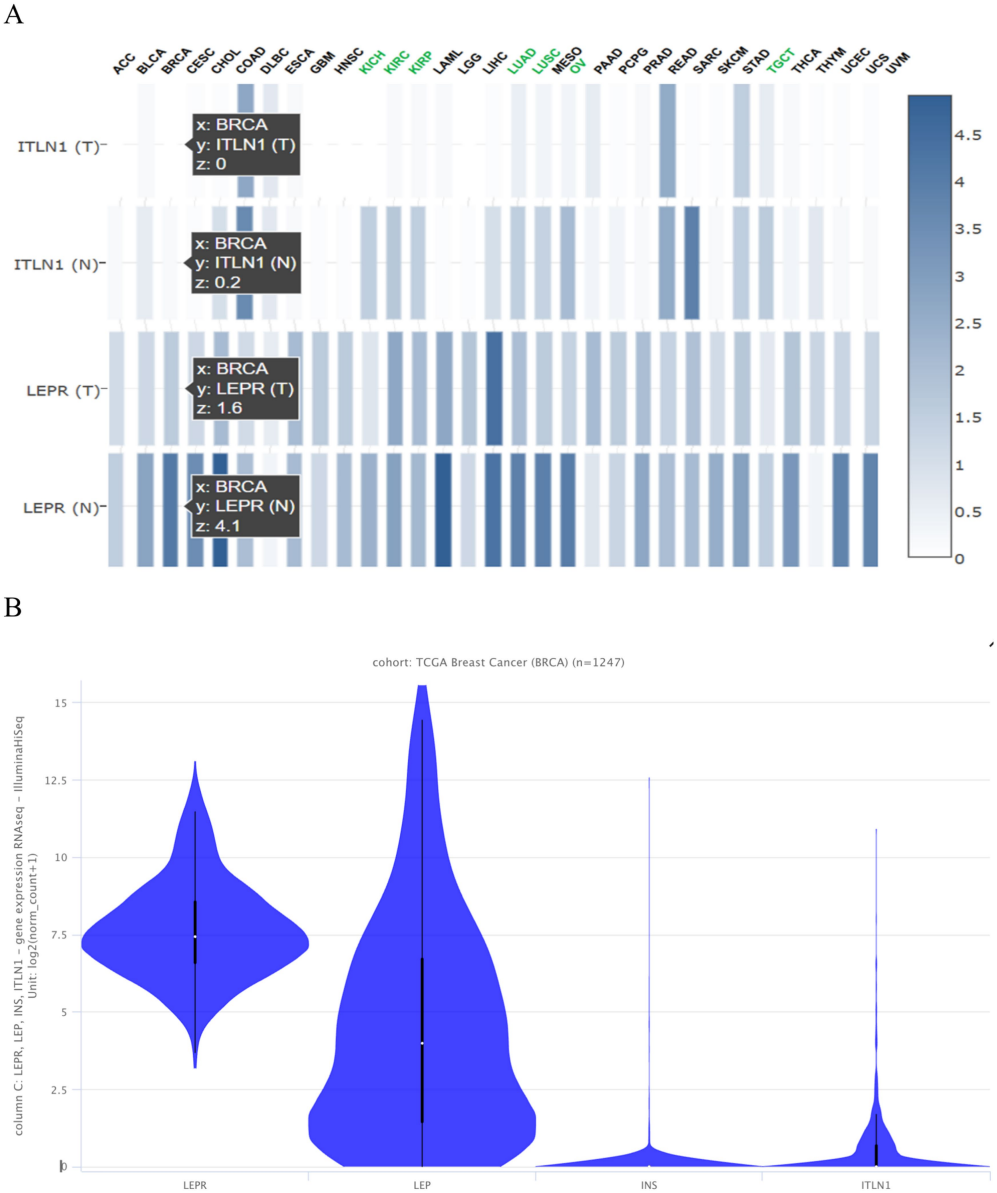
Expression of the tested genes **(A)** in different types of cancer presented as a heatmap from Gepia 2 and **(B)** in the TCGA BC cohort of 1,247 samples presented as a violin plot from https://xenabrowser.net/heatmap/.

Using the Gepia 2 gene expression profiling interactive analysis tool, CD295 and ITLN1 are found to be downregulated in different types of cancer, as shown in Figure 4A. Figure 4B for leptin, its receptor (CD295), ITLN1, and insulin in the BC cohort.

**Figure 5:**
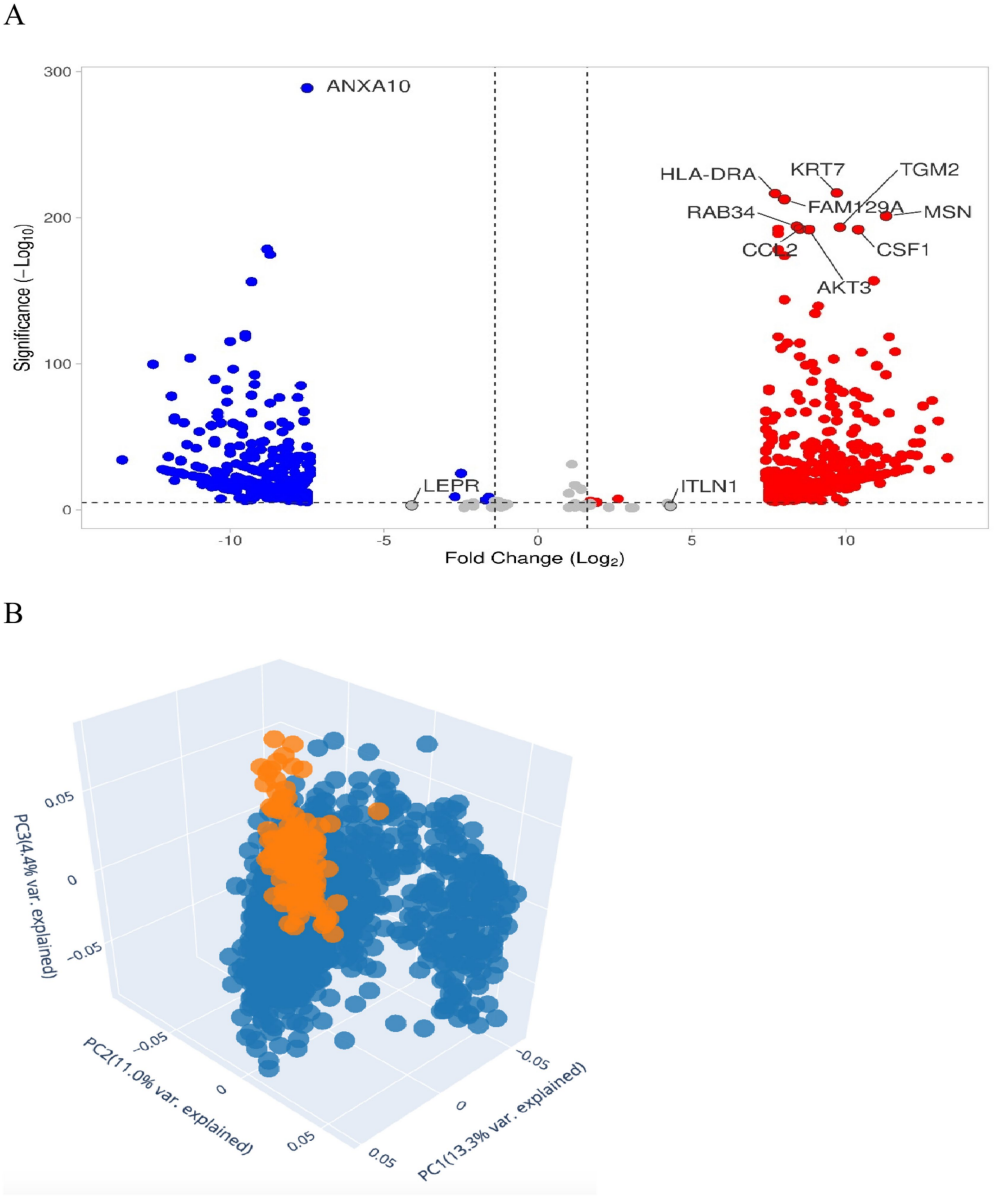
DEGs in breast cancer. **(A)** Volcano plot shows dataset downloaded from EMBL-EBI expression atlas or by Xenahub.net by UCSC Volcano plot for Solid Tissue Normal vs. Primary Tumor. The figure contains an interactive scatter plot that displays the log2-fold changes and statistical significance of each gene calculated by performing a differential gene expression analysis. Genes with logFC > 1.5 and *p*-value < 0.05 in red and genes with logFC < −1.5 and p-value < 0.05 in blue. **(B)** 3D PCA scatter plot of the samples’ data using 2,500 genes having the largest variance. Each point represents a sample of gene expression. Samples with similar gene expression profiles are closer in the three-dimensional space. Solid tissue appears normal, with an orange color compared to the primary tumor, which is blue.

While both CD295 (LEPR) and ITLN1 show no significant differential expression in tumor and normal cells when analyzing the microarray dataset from the EMBL-EBI expression atlas, as shown in Figure 5A. Supplementary Table S1 differentially expressed genes between solid tissue normal vs. primary tumor using limma voom from the http://analysis.xenahubs.net/c3f1c26960c418e75aeb1d2d6972d96008783fef/.

Figure 5B represents statistical PCA of the most relevant sources of variance in the data and is subsequently visualized using a scatter plot of 139 normal solid tissue vs. 1,101 primary tumors.

#### 3.7.2 CD295 and ITLN1 protein–protein interaction model by STRING

**Figure 6:**
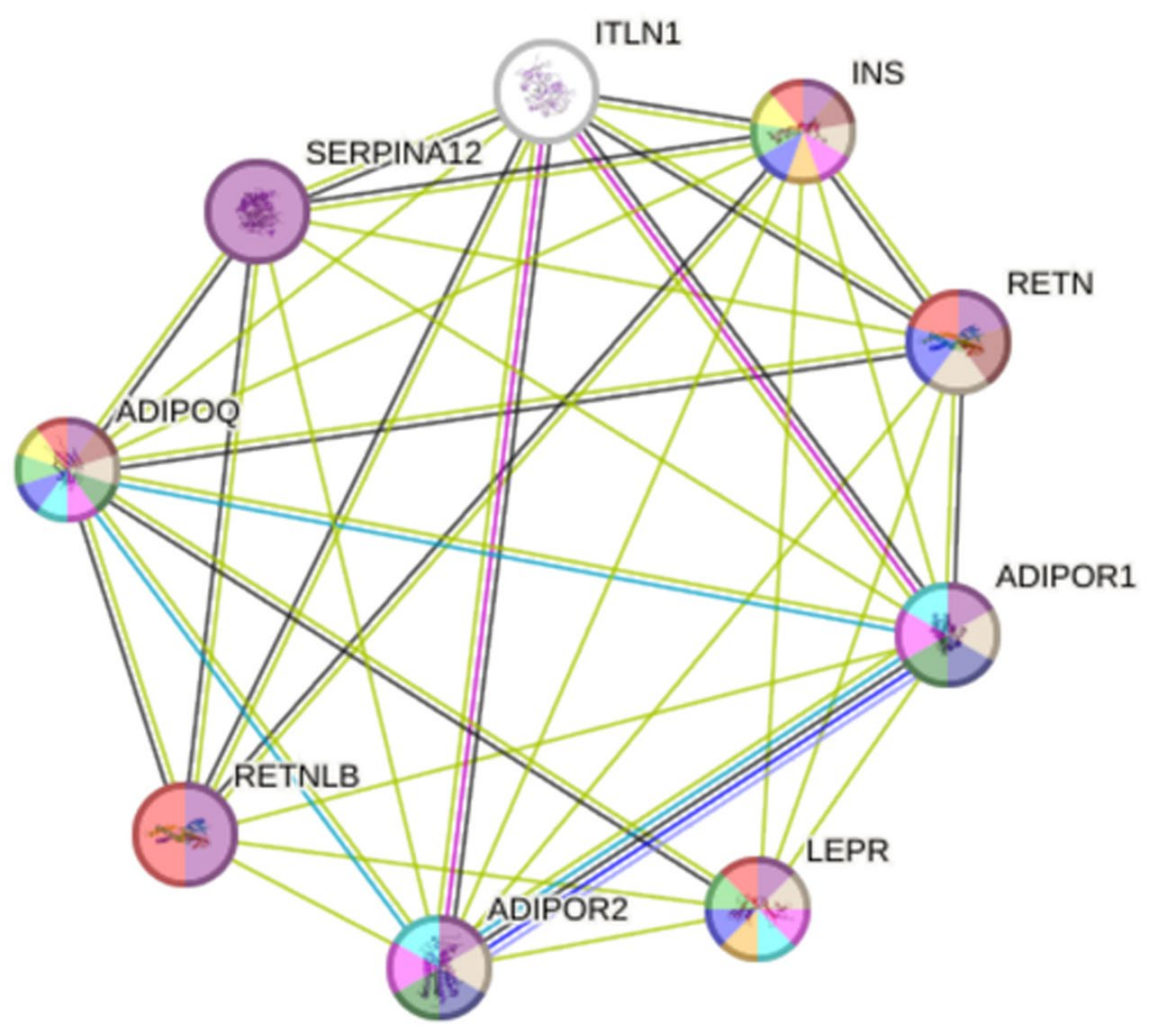
PPI network constructed using STRING database. ADIPOQ, Adiponectin; ADIPOR1 and 2, Adiponectin receptor 1 and 2; INS, Insulin; ITLN1, Intelectin-1; LEPR; Leptin receptor (CD295); RETN, Resistin; RETNLB, Resistin-like β; SERPINA12, Serpin A12.

Figure 6 shows a PPI network constructed by the STRING database, which includes our two studied proteins connected to the other seven nodes of the most interacted protein. This suggests that our proteins are linked to insulin resistance, signal transduction, and inflammation. Both LEPR (CD295) and ITLN1 are linked to insulin (INS), adiponectin, adiponectin receptor 1 and 2, resistin, and resistin-like *β* proteins. While only ITLN1 is linked to serpin A12, which is an insulin-action modulator adipokine.

CD295 is co-expressed with adiponectin with a score = 0.043. While ITLN1 is co-expressed with adiponectin receptor 1, resistin, and resistin-like β with co-expression scores = 0.058, 0.062, and 0.162, respectively, as shown in Table 7.

**Table 7:** Linkage and co-expression score between the studied protein and the most linked protein related to IR and BC.

Scores	CD295(LEPR)		ITLN1	
	Linkage	Co-expression	Linkage	Co-expression
Linked protein				
Insulin	0.87	–	0.592	–
Adiponectin	0.864	**0.043**	0.771	–
Adiponectin receptor 1	0.71	–	0.446	**0.058**
Adiponectin receptor 2	0.606	–	0.418	–
Resistin	0.606	–	0.773	**0.062**
Resistin-like β	0.728	–	0.772	**0.162**
Serpin A12	–	–	0.84	

All bold numbers indicate significant *p*-value.

#### 3.7.3 Enrichment analysis and gene-disease association results

The expanded subnetwork reveals several key associations from KEGG pathways: the gene product LEPR is involved in the Janus kinases (JAKs) and signal transducers and activators of transcription (STATs) JAK–STAT signaling pathway, the non-alcoholic fatty liver disease pathway, the cytokine-cytokine receptor interaction pathway, the adipocytokine signaling pathway, and the AMP-activated protein kinase (AMPK) signaling pathway.

**Figure 7:**
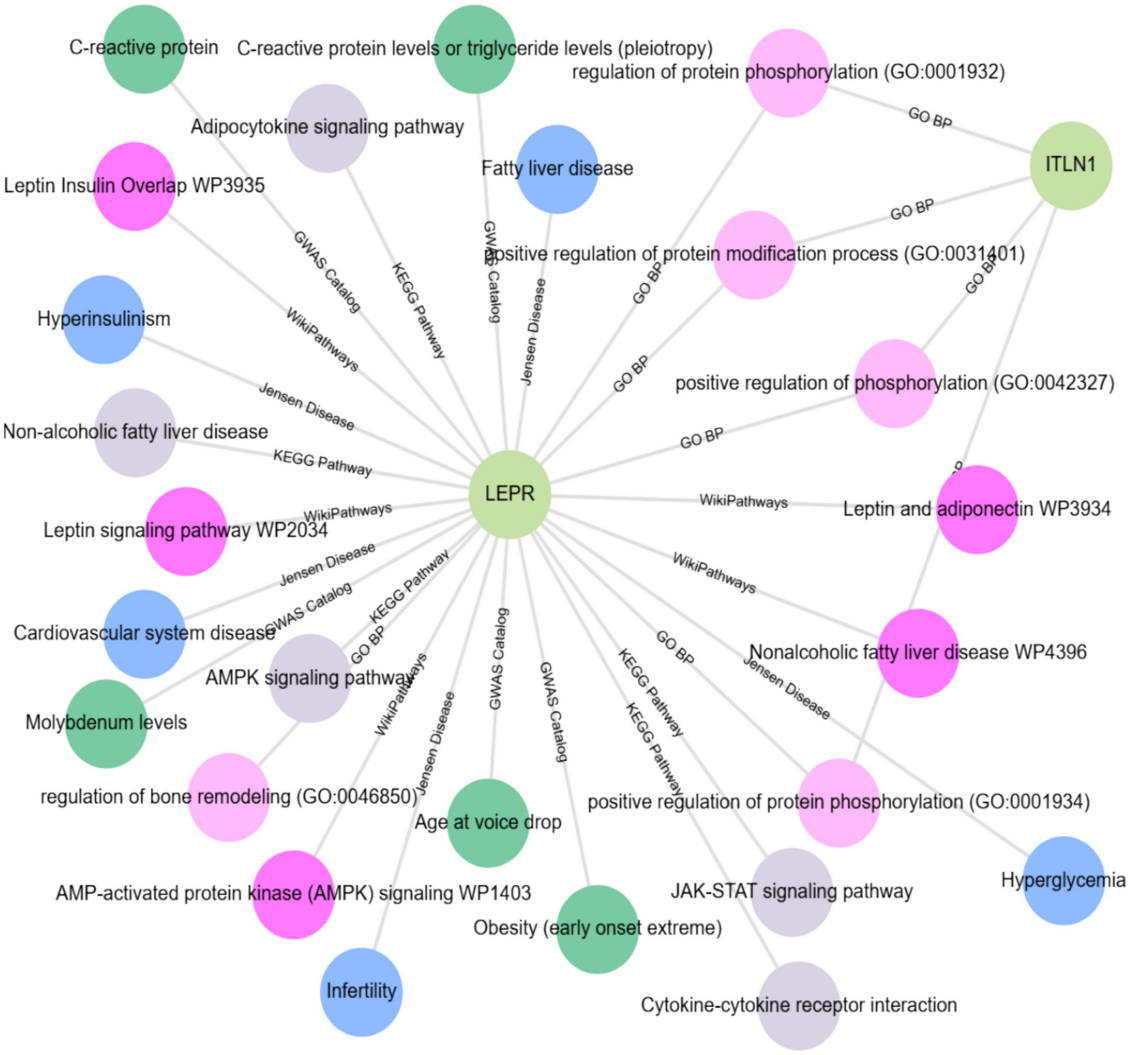
Enrichment analysis (EA) and gene-disease association (GDA) using the Enrichr-KG database accessed at https://maayanlab.cloud/enrichr-kg.

JensenLab^14^ has developed advanced databases and analytical tools for constructing and examining molecular interaction networks using both proteomics data and automated text mining techniques. The Enrichr-KG^15^ uses the Jensen lab database to augment the subnetwork, highlighting the associations of the LEPR gene with hyperinsulinism, fatty liver disease, infertility, cardiovascular system disease, and hyperglycemia. While using the genome-wide association studies (GWAS) catalog to link the LEPR gene with several traits and conditions, including C-reactive protein levels, molybdenum levels, age at voice drop, and pleiotropic effects on C-reactive protein and triglyceride levels, as well as early-onset extreme obesity. According to WikiPathways (WP),^16^ the LEPR gene is included in several pathways: Leptin and adiponectin (WP3934), Leptin Insulin Overlap (WP3935), Nonalcoholic fatty liver disease (WP4396), Leptin signaling pathway (WP2034), and the AMP-activated protein kinase (AMPK) signaling pathway (WP1403). According to Gene Ontology (GO),^17^ both LEPR and ITLN1 are involved in the biological processes of positive regulation of phosphorylation (GO:0042327), positive regulation of the protein modification process (GO:0031401), and regulation of protein phosphorylation (GO:0001932). Moreover, LEPR is associated with the regulation of bone remodeling (GO:0046850), and both LEPR and ITLN1 participate in the positive regulation of protein phosphorylation (GO:0001934). All these associations are shown in Figure 7.

#### 3.7.4 Genotype-tissue expression and genotype survival analysis results

**Figure 8:**
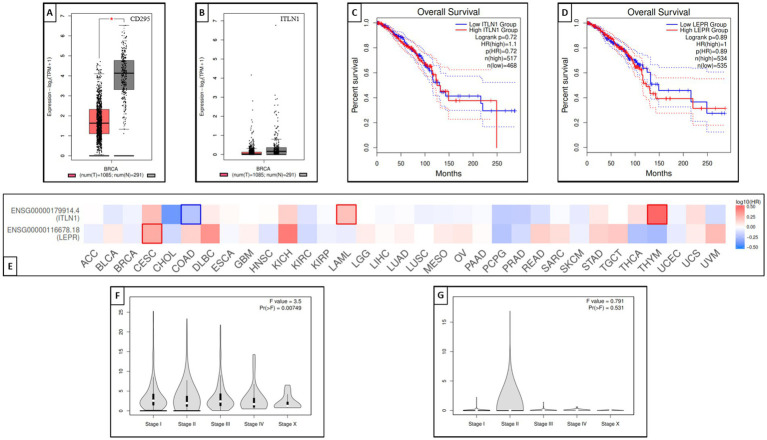
Genotype-tissue expression and genotype survival analysis; **(A)** differential expression of LEPR gene in BRCA and normal tissue, **(B)** differential expression of ITLN1 gene in BRCA and normal tissue, **(C)** overall survival percentage with high and low LEPR expression level, **(D)** overall survival percentage with high and low ITLN1 expression level, **(E)** survival map of LEPR and ITLN1 genes in different cancer types, **(F)** differential expression violin blot of LEPR gene in different BRCA stages, and **(G)** differential expression violon blot of LEPR gene in different BRCA stages. ACC, Adrenocortical carcinoma; BLCA, Bladder Urothelial Carcinoma; BRCA, Breast invasive carcinoma; CESC, Cervical squamous cell carcinoma and endocervical adenocarcinoma; CHOL, Cholangio carcinoma; COAD, Colon adenocarcinoma; DLBC, Lymphoid Neoplasm Diffuse Large B-cell Lymphoma; ESCA, Esophageal carcinoma; GBM, Glioblastoma multiforme; HNSC, Head and Neck squamous cell carcinoma; KICH, Kidney Chromophobe; KIRC, Kidney renal clear cell carcinoma; KIRP, Kidney renal papillary cell carcinoma; LAML: Acute Myeloid Leukemia; LGG, Brain Lower Grade Glioma; LIHC, Liver hepatocellular carcinoma; LUAD, Lung adenocarcinoma; LUSC, Lung squamous cell carcinoma; MESO, Mesothelioma; OV, Ovarian serous cystadenocarcinoma; PAAD, Pancreatic adenocarcinoma; PCPG, Pheochromocytoma and Paraganglioma; PRAD, Prostate adenocarcinoma; READ, Rectum adenocarcinoma; SARC, Sarcoma; SKCM, Skin Cutaneous Melanoma; STAD, Stomach adenocarcinoma; TGCT, Testicular Germ Cell Tumors; THCA, Thyroid carcinoma; THYM, Thymoma; UCEC, Uterine Corpus Endometrial Carcinoma; UCS, Uterine Carcinosarcoma; UVM, Uveal Melanoma.

GEPIA is a widely used and highly cited platform for analyzing gene expression, utilizing tumor and normal sample data from The Cancer Genome Atlas Program (TCGA) and Genotype-Tissue Expression (GTEx) databases. Proportion analysis was conducted for both genes and revealed that CD295 is significantly underexpressed in BC tissue when compared with normal breast tissue (Figure 8A), while there was no significant difference in ITLN1 gene expression between tumor and normal tissue (Figure 8B). The Kaplan–Meier (KM) curve serves as a standard tool for evaluating time-to-event outcomes, including survival time. The KM for percent survival with the level of gene expression shows that no significant difference in survival percent is associated with different expression levels of both genes (Figures 8C,D) in BC, but it may show a significant difference in other types of cancers, such as cervical squamous cell carcinoma and endocervical adenocarcinoma, colon adenocarcinoma, acute myeloid leukemia, and thymoma (Figure 8E). LEPR shows differential expression among breast invasive carcinoma stages, while ITLN1 shows differential expression in stage II only (Figures 8F,G).

## 4 Discussion

The results of the current study demonstrated, via biochemical and genotyping analysis supported by an *in silico*/bioinformatics search study, that the CD295 gene is highly polymorphic among BC patients. This analysis demonstrates compelling evidence of genetic-metabolic interactions in BC pathogenesis through multiple statistical approaches. We found that the CD295 polymorphism was associated with BC cases having a late TN classification, as well as FSH levels > 25 mIU/L and a HER2 score of +3.

Furthermore, BC patients who were obese, diabetic, or pre-diabetic were more likely to carry the type I cytokine receptor, LEPR, and CD295 polymorphism. Those patients who had CC or CT CD295 rs6700986 genotypes had a higher incidence of tumor clinical stage T II, positive LN involvement, histologic grade III, obesity, pre-diabetes, IR, or DM.

The CD295 protein is encoded by a gene belonging to the 130-member family of glycoprotein cytokine receptors that are known to enhance gene transcription by activating cytosolic STAT proteins (12). Furthermore, functioning in hematopoietic pathways that are required for lymphopoiesis, CD295 functions as a receptor for leptin, a hormone that acts on adipocytes to regulate body weight (31, 32). Indeed, mutations in the gene that encodes leptin have been related to an increased frequency of obesity (14). Moreover, leptin plays a role in cell growth and differentiation, as well as angiogenesis, by promoting increased production of signaling molecules such as NO, VEGF, FGF2, and VEGFR2 by endothelial cells (33).

In cancer, LEP is considered to act as both a mitogenic and migration factor in malignant cells (33). In the context of BC, however, Woo et al. (46) found no significant connection between four polymorphisms in the LEPR gene and the risk of BC. They likewise did not observe noteworthy differences in serum LEP levels between BC patients and controls.

Furthermore, we examined the emerging role of ITLN1 SNP in BC and found that the rs952804 polymorphism was related to BC risk indices (grade III, positive LN involvement) as well as to the presence of obesity, IR, DM, and pre-diabetes. These findings are consistent with earlier reports showing that ITLN1 participates in immune defense and insulin-stimulated glucose uptake in human subcutaneous and omental adipocytes (16). In particular, BC cases that were CT for the ITLN1 rs952804 mutant (CT) were significantly associated with tumor clinical stage T II and positive LN involvement, as well as tumor histologic grade III, presence of obesity, pre-diabetic event, DM, and IR (*p* ≤ 0.05).

Relative to the adipokine vaspin, ITLN1 levels appear as superior indicators of IR among obese patients in the study group by Sperling et al. (34, 35).

Clinical significance of the correlation heatmap for these effect sizes represents substantial genetic risk factors, with the C allele of rs6700986 and the T allele of rs952804 showing strong associations with BC susceptibility. The magnitude of these associations suggests clinically meaningful genetic variants that could inform risk stratification.

Obesity is an important health problem and is positively correlated with the occurrence and mortality of BC (33). Moreover, obese BC patients are known to have a higher risk of LN metastasis, larger tumors (36, 37), and higher death rates compared with non-obese patients (38). These increased risks might be due to elevated estrogen levels that occur from enhanced aromatization in the adipose tissue and increases in the levels of mitogenic agents such as INS and/or IGF associated with obesity-related metabolic syndromes (DM and BC) (18).

Beyond increased estrogen levels, increases in INS levels may also contribute to the development of BC by enhancing insulin-like growth factor-I (IGF-1) receptor-mediated release of VEGF from breast tissues (39). This could be coupled with the effects associated with increased LEP levels (33). Moreover, the adverse influence of DM on the prognosis of cancer patients is likely associated with the effects of INS on the tyrosine kinase growth receptor pathway (19).

INS, IGF-I, and hybrid IGF-I/INS receptors are all overexpressed in BC cells (40). As such, activation of these receptors could upregulate the expression of INS receptor substrate 2, which in turn activates downstream MAPK and phosphatidylinositol 3-kinase-Akt pathways (19). Indeed, these speculations were confirmed by bioinformatics analysis.

Indeed, *in vitro* studies have demonstrated that ITLN1 increases INS signal transduction by activating the protein kinase Akt/protein kinase B signaling and enhancing insulin-stimulated glucose transport in isolated human adipocytes. Increased BC risk related to hyperinsulinemia could be attributed to a synergistic interaction between elevated free estrogen concentrations and aberrant INS signaling (41, 42). Furthermore, the ability of ITLN1 to activate AKT, a key survival factor, could be a mechanism for ITLN1-mediated cell proliferation in BC (16). This effect, together with CD295 stimulation of gene transcription associated with activation of cytosolic JAK–STAT proteins, MAPK, PI3K, and AMPK (14), may contribute to the increased frequency of ITLN1 and CD295 polymorphisms seen in the current study.

It should be noted that ITLN1 rs952804 mutant CT, specifically the T allele, was related to BC risk, DNA damage, obesity, DM, and IR. Furthermore, there was no statistically significant difference between our observations and those predicted by the Hardy–Weinberg principle for ITLN1, suggesting that the population was in HWE (data not shown). Thus, the null hypothesis is not rejected, and we presume that the population is indeed in HWE, although additional study is warranted for this locus with the metastatic population (future perspective).

The Kaplan–Meier analysis did not reveal statistically significant differences in survival based on CD295 or ITLN1 gene expression. This finding suggests that single-gene expression levels may not be sufficient to stratify prognosis in isolation. Contributing factors may include biological heterogeneity, limited statistical power, and the follow-up duration. Integrative models combining clinical, molecular, and genetic data may enhance prognostic accuracy.

While CD295 and ITLN1 expression did not correlate with survival outcomes in our analysis, these genes are involved in key pathways linked to cancer metabolism, inflammation, and immune modulation. Prior research has associated CD295 with insulin signaling and adipokine regulation, while ITLN1 plays a role in innate immunity. The lack of survival association in our cohort may reflect context-specific gene activity or limitations in our sample.

Although the observed associations between SNP genotypes and DNA tail damage reached statistical significance, their clinical predictive value remains uncertain. Diagnostic metrics, such as the area under the curve (AUC), sensitivity, and specificity, were not calculated due to the study’s sample size and exploratory design.

The subgroup analyses (e.g., for IR, DM, and obesity) were exploratory and not sufficiently powered *a priori*. Thus, these findings should be interpreted with caution and warrant validation in larger, stratified cohorts. Future studies should include stratified power calculations to ensure adequate sensitivity for subgroup-level analyses.

A key strength of this research is its adherence to modern statistical reporting guidelines; our findings emphasize both statistical significance and effect sizes. The correlation coefficients, odds ratios (inferred from genotype frequency differences), and group differences in biomarker levels all suggest clinically meaningful associations rather than merely statistical artifacts. However, the varied populations and population stratification, which were previously a confounding limiting factor, are not in the current study.

### 4.1 Limitation

Analyzing combinations of genetic variants (haplotypes) can provide a more powerful assessment of disease risk compared to studying single SNPs alone. For more definitive causal results, rather than associative, the current potential susceptibility markers are pending validation in larger and more diverse cohorts.

## 5 Conclusion

It is possible that polymorphisms of CD295 or potentially ITLN1 could contribute to variations in BC occurrence. Nonetheless, in this study, SNPs in CD295 and ITLN1 were related to oxidative stress/DNA damage in postmenopausal Egyptian female BC patients.

This study showed an increased risk of BC in women carrying the C allele of the CD295 rs6700986 polymorphism and the T allele of the ITLN1 rs952804 polymorphism. According to graphical abstract, biochemical assays and genotyping analysis supported by an *in silico*/bioinformatics study, as well as enrichment and survival analysis, suggest that CD295 rs6700986 and ITLN1 rs952804 SNPs could be considered, together with DNA damage, as BC-related risk factors.

Moreover, we have to acknowledge the exploratory nature of multiple comparisons while highlighting the biological coherence of the findings. Additionally, to emphasize the integrated nature of genetic-metabolic interactions rather than individual marker effects. ITLN1 and CD295 polymorphism testing could be used to assess BC susceptibility in either obese or insulin resistance, pre-diabetic patients. We therefore suggest that this high-risk BC population be screened for these SNPs.

### 5.1 Recommendations

Broaden the research scope to include more metabolic factors associated markers, such as lipocalins (43) or hypoxia inducible factor-1 (44) SNPs in relation to DNA damage with ITLN1 and CD295 in BC in either obese or insulin resistance, pre-diabetic patients.

## Data Availability

The original contributions presented in the study are included in the article/Supplementary material, further inquiries can be directed to the corresponding author.

## Footnotes

^1^ https://thebiogrid.org/110144/table/homo-sapiens/lepr.html

^2^ https://ctdbase.org/detail.go?type=gene&acc=3953&view=go

^3^ https://thebiogrid.org/120741/summary/homo-sapiens/itln1.html

^4^ https://ctdbase.org/detail.go?type=gene&acc=55600&view=disease

^5^ http://www.ncbi.nlm.nih.gov/snp/?term

^6^ https://www.instem.com/solutions/study-management/comet-assay-iv/

^7^ https://www.ebi.ac.uk/gxa/home

^8^ https://huygens.science.uva.nl/VolcaNoseR/

^9^ https://xenabrowser.net/

^10^ https://string-db.org/

^11^ http://gepia.cancer-pku.cn/

^12^ https://www.statskingdom.com/shapiro-wilk-test-calculator.html

^13^ https://www.had2know.org/academics/hardy-weinberg-equilibrium-calculator-2-alleles.html

^14^ https://jensenlab.org/

^15^ https://maayanlab.cloud/enrichr-kg

^16^ https://www.wikipathways.org/

^17^ https://geneontology.org/

## Glossary

ABI: Applied Biosystems International
ADIPOQ: Adiponectin
ADIPOR1 and 2: Adiponectin receptor 1&2
AMPK: AMP-activated protein kinase
BC: breast cancer
BI-RADS: breast imaging-reporting and data system
CA15.3: cancer antigen 15.3
CD: cluster of differentiation
CEA: carcinoembryonic antigen
DEGs: differentially expressed genes
EA: Enrichment Analysis
ER: estrogen receptor
FSH: follicle stimulating hormone
GDA: Gene-Disease Association
GEPIA: Gene Expression Profiling Interactive Analysis
GO: Gene Ontology
GTEx: Genotype-Tissue Expression
GWAS: genome-wide association studies
HER2: human epidermal receptor 2
HOMA-B: HOMA of *β*-cell function
HOMA: Homeostasis model assessment
HWE: Hardy–Weinberg equilibrium
IC: informed consent
IDC: invasive ductal carcinoma
INS: insulin
IR: insulin resistance
ITLN1: adipocyte-inferred cytokine intelectin 1
JAK: Janus kinases
KM: Kaplan–Meier
LEP: leptin
LEPR: leptin receptor
LH: luteinizing hormone
n: number
NCI: National Cancer Institute
NS: non-significant
OR: odds ratio
PCA: Principal Component Analysis
PCR: polymerase chain reaction
PCs: Principal Components
PPI: Protein–protein interaction
PR: progesterone receptor
R: receptor
RETN: Resistin
RETNLB: Resistin-like β
ROC: receiver operating characteristics
SD: standard deviation
SERPINA12: Serpin A12
SNPs: single-nucleotide polymorphisms
SPSS: statistical package for social sciences
STAT: signal transducers and activators of transcription
TCGA: The Cancer Genome Atlas Program
TI: tail intensity
TMI: tail moment unit
TMN: tumor-metastasis-node
UCSC: University of California Santa Cruz
WP: WikiPathways
